# Strengthening Routine Immunization in Areas of Northern Nigeria at High Risk for Polio Transmission During 2012–2014

**DOI:** 10.1093/infdis/jiv580

**Published:** 2016-04-02

**Authors:** Daniel Ali, Richard Banda, Abdulaziz Mohammed, Julie Adagadzu, Bolatito Murele, Rachel Seruyange, Jeevan Makam, Pascal Mkanda, Bassey Okpessen, Sisay G. Tegegne, Adeboye S. Folorunsho, Tesfaye B. Erbeto, Yared G. Yehualashet, Rui G. Vaz

**Affiliations:** 1 World Health Organization, Country Representative Office; 2 National Primary Health Care Development Agency, Abuja, Nigeria; 3 World Health Organization, Regional Office for Africa, Brazzaville, Congo

**Keywords:** routine immunization, intensification, polio, high risk, local government areas, supportive supervisory visits

## Abstract

***Background.*** Following the 2012 declaration by World Health Organization (WHO) Regional Director for Africa and the WHO Executive Board to ramp up routine immunization (RI) activities, began to intensify activities to strengthen RI. This study assessed how the intensification of RI helped strengthen service delivery in local government areas (LGAs) of northern Nigeria at high risk for polio transmission.

***Methods.*** A retrospective study was performed by analyzing RI administrative data and findings from supportive supervisory visits in 107 high-risk LGAs.

***Results.*** Our study revealed that administrative coverage with 3rd dose of diphtheria-pertussis-tetanus vaccine in the 107 high-risk LGAs improved from a maximum average coverage of 33% during the preintensification period of 2009–2011 to 74% during the postintensification period of 2012–2014.

***Conclusions.*** Routine immunization could be strengthened in areas where coverage is low, and RI has been identified to be weak when certain key routine activities are intensified.

Despite global efforts, the routine immunization (RI) coverage in 2 World Health Organization (WHO) regions, namely the African Region and the South-East Asia Region, still remained short of 2010 targets [1]. As a result, the WHO Regional Director for Africa declared 2012 as the year for intensifying RI in the region; this also coincided with the declaration of polio as a programmatic emergency by the WHO Executive Board in 2012.

Furthermore, the Independent Monitoring Board (IMB) Report of November 2012 also alluded to the need to ramp up RI in states at high risk for polio, such as Kano [2]. The 22nd and the 23rd Nigerian Expert Review Committee on polio and RI in 2012 recommended the intensification of RI activities, such as the 1, 2, 3 strategy (in which all health facilities conduct at least 1 fixed session every week, each health facility conducts at least 2 outreach sessions in a month, and the local governmental area [LGA] team members conduct at least 3 supervisory visit each month to health facilities), and this was included in the National Polio Eradication Emergency Plan 2012 [3–5].

The challenges and barriers to achieving high immunization coverage in Nigeria include inadequate planning, poor implementation of planned activities, inadequate supportive supervision, lack of monitoring for action, inadequate involvement of the community in immunization activities, and poor funding of planned RI activities [6]. The ways of addressing these barriers include intensifying RI activities and improving operational efficiency.

In 2012, Nigeria began to intensify activities to strengthen RI. These intensified activities include training of health workers on RI, updating Reaching Every Ward microplans, increasing immunization sessions (fixed and outreach sessions), intensifying supportive supervision to the LGA and health facilities, increasing demand creation for RI services, ensuring vaccine availability (through stock monitoring and distribution of vaccines), monitoring for action (use of data for action), and leveraging immunization-plus days, local immunization days, and African Vaccination Week as conduits to provide additional RI services.

Previous work closely related to this study include research by Closser et al [7] and Gidado et al [8]. The former study used a combination of qualitative and quantitative methods to examine the relationship between Polio Eradication Initiative activities, RI, and PHC in 7 countries from Asia and sub Saharan Africa. This study from the qualitative findings showed positive impact of PEI on disease surveillance and cold chain strengthening. The study by Gidado et al only identified households and settlements missed by polio teams during polio campaigns using site visits and interviews. We conducted this study to document the contribution of the intensification of RI to strengthening service delivery at LGA and health facilities in northern Nigeria.

## METHODS

### Study Design

We conducted a retrospective study, using a literature review of previous assessments and data generated from the RI administrative database and supportive supervisory visits to health facilities in 107 LGAs at very high risk for polio transmission.

### Study Population

In 2012, the National Emergency Operation Center, which coordinates all polio eradication activities in Nigeria, identified 107 LGAs that were at a high risk for polio transmission. The criteria used by the National Emergency Operation Center in arriving at these LGAs were evidence of wild poliovirus, evidence of circulating vaccine-derived polioviruses, supplemental immunization activity (SIA) coverage, and RI coverage [9].

### Data Collection

Standard RI data management tools were distributed to all LGAs, including those at high risk for polio transmissions. Health workers received training on the revised data management tools. Data were collected using health facility tally sheets and are summarized at the LGA level on a monthly basis and transmitted to the national level through the states and zones. A system of data quality checks to clean up the data at the state and zonal levels has been institutionalized and used. Supportive supervision data were collected using short messaging service and mobile data devices on a Magpi platform (Magpie Website Solutions ©2014), which is a mobile data collection platform [10]. WHO staff, including surge capacity personnel, visited health facilities on a weekly basis and filled in the data, which were transmitted on the Magpi platform. The data were then downloaded and analyzed at the national level, and feedback was sent to the states. A desk review of these in-country data was conducted during the study, and the data reviewed for this study were RI 3rd dose of diphtheria-pertussis-tetanus vaccine (DPT3) coverage and 3rd dose of oral polio vaccine (OPV3) coverage obtained from the District Vaccine Data Management tool (DVD-MT) for administrative data, Nigeria Vaccine Audit Report of 2012 [11], and supportive supervisory findings for the107 LGAs for 3 years before (ie, 2009–2011) [12–14] and 3 years into (ie, 2012–2014) [15–17] the RI intensification program. The data sets from 2009–2011 were reviewed and compared to data sets from 2012–2014 for the 107 LGAs.

### Intervention

The interventions that were provided to intensify RI in the high-risk LGAs beginning in 2012 included training of health workers on RI and other Expanded Program on Immunization components, such as surveillance, communication, and logistics; updating Reaching Every Ward microplans that link with SIA microplans; increasing the immunization sessions (fixed and outreach sessions); intensifying supportive supervision to the LGAs and health facilities; monitoring fixed and outreach sessions; increasing demand creation for RI services; ensuring vaccine availability through stock monitoring and supporting distribution of vaccines to LGAs, health facilities, and outreach sites; monitoring for action (including updating of monitoring charts and use of data for action); and leveraging immunization-plus days, local immunization days, maternal and newborn child health weeks, and African vaccination weeks as strategies to provide RI services.

### Variables

Administrative and supervisory data from 2009–2011 (ie, before RI intensification) were compared to the same data set from 2012–2014 data (ie, after RI intensification) in the 107 LGAs.

### Data Analysis

These interventions were analyzed in terms of the process indicators and output indicators during 2010–2013, using Microsoft Excel, to demonstrate trend over the years.

## RESULTS

### Supportive Supervisory Findings

The proportion of planned integrated supportive supervisory visits that were conducted by WHO staff (state coordinators, cluster coordinators, and LGA facilitators) was 23% in March 2014 and 87% in December 2014. A total of 13 590 supportive supervisory visits (70%) were conducted out of 19 404 supervisory visits planned from March to December 2014 (Table 1).

**Table 1.** Integrated Supportive Supervision (ISS) Activities Planned and Conducted by World Health Organization Field Personnel, March–December 2014

ISS Activities	March	April	May	June	July	August	September	October	November	December	Total
Planned, no.	132	1077	1482	1635	1983	2286	2646	2547	2913	2703	19 404
Conducted, no. (%)	31 (23)	446 (41)	1045 (71)	613 (37)	1210 (61)	1631 (71)	1801 (68)	2133 (84)	2329 (80)	2351 (87)	13 590 (70)

Data are from the World Health Organization Nigeria mobile data device database 2014.

The proportion of planned fixed sessions (FS) and outreach sessions (OS) conducted in the 107 LGAs was 81% and 63%, respectively, in March 2014 and 97% and 93%, respectively, in December 2014. Fewer than 80% of planned FS were conducted in April 2014, and fewer than 80% of planned OS were conducted during March–May 2014 (Table 2).

**Table 2.** Proportion of Planned Fixed and Outreach Sessions Conducted in Health Facilities in 107 Local Government Areas at High Risk for Polio Transmission, March–December 2014

Variable	March	April	May	June	July	August	September	October	November	December
Fixed, %	81	68	94	96	96	93	93	97	94	97
Outreach, %	63	62	79	86	83	85	90	91	89	93

Data are from the World Health Organization Nigeria mobile data device database 2014.

**Figure 1.**
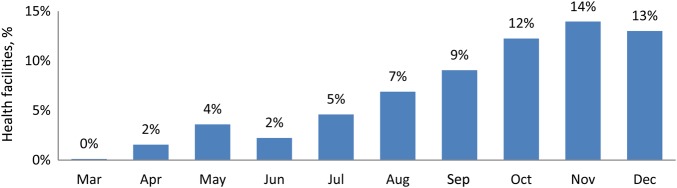
Trends of proportion of health facilities with defaulter tracing, March–December 2014. Data are from WHO Nigeria mobile data device database 2014.

In the 107 LGAs, the proportion of health facilities that had defaulter tracking systems in place or institutionalized was 0% in March 2014 and 13% in December 2014 (Figure 1).

**Figure 2.**
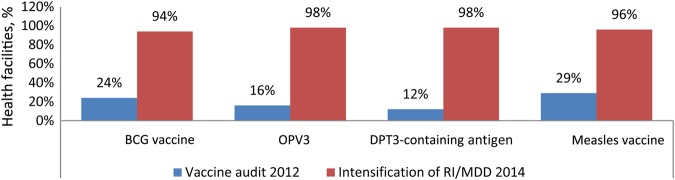
Comparison of the proportion of health facilities visited with adequate vaccine supply between the vaccine audit report of 2012 and the Nigeria mobile data device (MDD) database for 2014. Abbreviations: DPT3, 3rd dose of diphtheria-pertussis-tetanus vaccine; OPV3, 3rd dose of oral polio vaccine; RI, routine immunization.

The vaccine audit report of 2012 revealed that <30% of health facilities had BCG vaccine, OPV3, DPT3 and measles vaccine available for RI services. In 2014, the supportive supervisory findings from the 107 high-risk LGAs indicated that >90% of the health facilities had BCG vaccine, OPV3, DPT3, and measles vaccine available (Figure 2).

### Administrative Coverage

The proportion of the 107 high-risk LGAs that achieved DPT3 coverage of ≥80% was 23% in 2009 and 74% in 2014. The proportion of LGAs that achieved OPV3 coverage of ≥80% was 6% in 2009 and 78% in 2014. An improvement in DPT3 and OPV3 coverage in 2013 and 2014 was observed in the LGAs following commencement of intensification of RI in 2012 (Table 3).

**Table 3.** Proportion of 107 Local Government Areas at High Risk for Polio Transmission That Achieved Third Dose Diphtheria-Pertussis-Tetanus Vaccine (DPT3) and Third Dose Oral Polio Vaccine (OPV3) Coverage of ≥80%, 2009–2014

Indicator	Before RI Intensification			During RI Intensification		
	2009	2010	2011	2012	2013	2014
DPT3 coverage ≥ 80%, no. (%)	25 (13)	35 (33)	22 (21)	13 (12)	65 (61)	79 (74)
OPV3 coverage ≥ 80%, no. (%)	6 (6)	18 (17)	17 (16)	28 (26)	44 (41)	83 (78)

Data are from the Nigeria District Vaccine Data-Management Tool 2009 to 2014.

Abbreviation: RI, routine immunization.

## DISCUSSION

We found that the maximum average DPT3 coverage in the 107 LGAs at high risk for polio transmission improved from 33% before the intensification of RI activities (during 2009–2011) and 74% after intensification. A similar improved trend was also observed in OPV3 coverage, with a maximum average OPV3 coverage of 17% before intensification and 78% after intensification. The dip in average DPT3 coverage of 12% in 2012, which was the year intensification of RI began in the 107 LGAs, was due to the prolonged nation-wide stock out of DPT vaccine as a result of the switch from DPT3 to pentavalent vaccine (which is a new DPT formulation) in 2012. However, the DPT situation improved in these LGAs in 2013 and 2014, as seen in the reported administrative coverage of 61% and 74% respectively.

We also found that the process indictors obtained from the supportive supervisory findings analyzed for 2014 for the 107 high-risk LGAs had improved. These process indicators measured the intensified RI performed to strengthen RI in these LGAs. The process indicators, which were monitored monthly, signified that RI sessions were adequately planned for by personnel in 86% of health facilities and that access to and use of immunization services by the communities improved in 67% of health facilities. Improvement in the conduct of planned FS and OS, from 81% and 63%, respectively, in March 2014 to 97% and 93%, respectively, in December 2014 connotes that virtually all planned sessions were performed, thereby reaching more children aged <1 year in the communities served by these facilities. This improvement was complemented by increased availability of routine vaccines at immunization sessions, from <30% of health facilities, as documented in a vaccine audit report of 2012, to >90%, based on supportive supervisory finding in 2014 . The number of health facilities that had a defaulter tracking system in place improved from 0% in March 2914 to 13% by December 2014. Also, the number of supportive supervisory visits conducted in these 107 LGAs improved from 23% in March 2014 to 87% in December 2014. This is evidence that more attention was given to the high-risk LGAs by WHO field personnel to ensure that intensified activities were done to strengthen RI in the LGAs. However, we discovered that there was no systematic way of documenting the findings from supportive supervision at the state and national levels until 2014. This coincided with the development of the WHO Nigeria integrated supportive supervision mobile data device platform to document the activities of WHO staff in conformity with the accountability framework.

Although the study demonstrated the contribution of intensified activities in strengthening RI in the targeted high-risk LGAs, there were inadequate data on supportive supervision between 2009 and 2013 to compare the trend of some of the key process indicators that the study set out to review. Despite this limitation, the findings from supportive supervisory visits during 2014 suggest that the contribution of intensified RI activities that commenced since 2012 could be a contributory factor that led to the improved DPT3 and OPV3 coverage observed in 2013 and 2014 in these LGAs. These improvements will lead to increased herd immunity against vaccine-preventable diseases, such poliomyelitis, among children.

We recommend that intensification of RI activities in other areas where coverage is low and RI has been identified as weak should be considered but that a sustainable mechanism should be elaborated while continuing and scaling up the implementation of these activities. We also recommend a study to compare the contribution of RI intensification in high-risk LGAs to that in non–high-risk homogenous LGAs.
